# Near-Field Communication Sensors

**DOI:** 10.3390/s19183947

**Published:** 2019-09-12

**Authors:** Zhonglin Cao, Ping Chen, Zhong Ma, Sheng Li, Xingxun Gao, Rui-xin Wu, Lijia Pan, Yi Shi

**Affiliations:** School of Electronic Science and Engineering, Nanjing University, Nanjing 210000, China; zhonglinCao555@163.com (Z.C.); chenping@nju.edu.cn (P.C.); mazhongnj@163.com (Z.M.); lisheng_nju@163.com (S.L.); njuxxgao@163.com (X.G.); rxwu@nju.edu.cn (R.-x.W.)

**Keywords:** near-field communications (NFC), sensors, energy harvesting, NFC antennas, wearable electronics, applications

## Abstract

Near-field communication is a new kind of low-cost wireless communication technology developed in recent years, which brings great convenience to daily life activities such as medical care, food quality detection, and commerce. The integration of near-field communication devices and sensors exhibits great potential for these real-world applications by endowing sensors with new features of powerless and wireless signal transferring and conferring near field communication device with sensing function. In this review, we summarize recent progress in near field communication sensors, including the development of materials and device design and their applications in wearable personal healthcare devices. The opportunities and challenges in near-field communication sensors are discussed in the end.

## 1. Introduction

With the advent of the Internet-of-Things (IoT) in the 21st century, wearable electronics technology will play critical roles in diverse fields of healthcare, food safety, security, etc. [1]. Integrating wearable devices with wireless communication data transmission provides new functions of real-time monitoring and dynamic response. Wireless communication technologies, such as radio frequency identification (RFID) and Bluetooth, have been widely used in wearable electronics for information transmission [2]. Among them, near-field communication (NFC) based on the RFID standards has attracted great attention due to its high security and simple operation process. NFC is a promising tool for physiological signal measurement with features such as battery-less in passive mode, wireless communication, fast and non-contact data transmission [3,4,5]. It has achieved successful applications in commercial consumption field by providing convenient payment methods, which gradually replaced the traditional way of paying with banknotes [6]. Compared with other RFID technologies (e.g., the ultrahigh frequency (UHF) RFID, and the chipless RFID), one difference is that it usually refers to the wireless device communicating in a short distance of less than 10 cm at 13.56 MHz [7,8,9]. Another difference is that peer-to-peer (P2P) communication can be achieved between the smartphone and NFC tags, while other RFID technologies are unable to realize this [10]. And unlike the Bluetooth, NFC tags can be battery-less, which promotes the development of low-cost devices.

In NFC electronics design, energy supply usually requires special consideration. The novel design concept endows NFC chips with capability of energy harvesting. These chips can collect the energy produced by the magnetic field generated by readers to provide an analog voltage output that can be used to power external electronics such as sensors. Some excellent commercial NFC chips integrated circuit (IC) with energy harvesting capability can be available in the market, facilitating the development of low-cost wearable devices [11]. Additionally, the design, materials, and manufacturing techniques of NFC antennas determine the performance of NFC devices. Different materials and manufacturing techniques of NFC antennas may lead the change of quality (Q) factors and read range of the devices, thus some special consideration requires to be applied to NFC antenna design.

**Figure 1:**
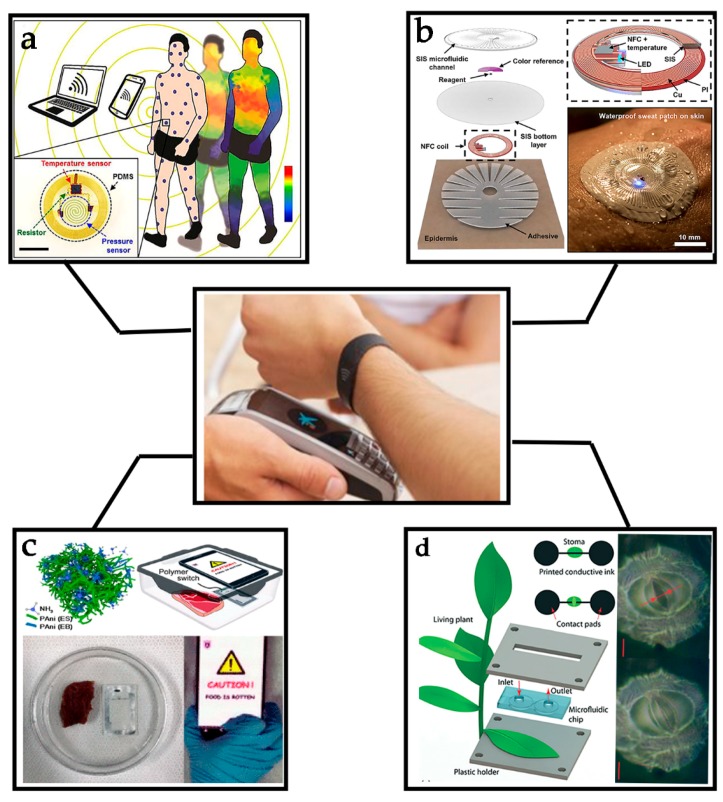
Recent progress in near-filed communication (NFC) sensors. (**a**) Biophysical signals monitoring. Reprinted with permission from ref. [21]. (**b**) Biochemical signals monitoring. Reprinted with permission from ref. [22]. (**c**) Food safety monitoring. Reprinted with permission from ref. [23]. (**d**) Plant growth monitoring. Reprinted with permission from ref. [24].

Nowadays, most smartphones are equipped with NFC modules, which provides a strong base to expand its scope of applications in human activities [12]. The combination of NFC tags with sensors becomes a new route to realize wireless communication sensed functions, which endows a smartphone with capabilities to rapidly obtain sensing information by simply reading an NFC tag integrated with a sensor [13]. Some approaches to using NFC technology have been developed to interface external sensors with smartphones [14]. These NFC sensors have been widely used in healthcare. As is shown in the Figure 1, NFC sensors have many other applications in continuous monitoring of food safety and plant growth, which shows great potential in commercial fields [15,16]. For example, in wearable devices, biochemical NFC sensors detecting biofluids (e.g., sweat, tears, and saliva) provide electronics with new functions in portable, real-time monitoring, non-invasive detection, and quantification [17,18,19,20]. 

In this review, NFC antennas and IC with the capability of energy harvesting are introduced to demonstrate the progress of NFC sensors. Several materials and manufacturing techniques are discussed to explore the potential of printing technologies for the NFC antennas. Some excellent representative devices equipped with NFC sensors are selected to illustrate the wide range of potential applications, for example, healthcare, food safety, and plant growth. The perspective of NFC sensors is discussed in the end.

## 2. Design of NFC Antennas

NFC chips with the capability of energy harvesting can facilitate the development of low-cost wearable devices. Meanwhile, the suitable design, materials, and manufacturing of NFC antennas can improve the performance. In this section, we will briefly introduce a comparison of wireless communication technologies and the fabrication of NFC antennas.

### 2.1. Comparison of Wireless Communication Technologies

Table 1 shows features of several kinds of RFID technologies and Bluetooth. Ultrahigh frequency (UHF) RFID technologies have been applied into the market, and several commercial sensors have been developed in logistics, inventory, and supply-chain applications [25,26,27]. Passive UHF RFID tags have the great capabilities of longer operation distance and low-cost than low frequency RFID tags [28]. However, the UHF readers are much expensive ($1000–$2000), which lead the difficulties in profit of commercial products [29]. On the other hand, UHF are more susceptible to environmental influence leading the losses and detuning [30]. Thus, a special design should be introduced to improve their performance [31,32]. Another representative RFID technology is chipless RFID, which has short read range (1–2 cm) for the frequency code and can reach 2–3 m for the time-domain-coded tags [33]. The cost of RFID devices is mainly dependent on the tags, which is the cost of chips. Thus, many efforts have been made to develop chipless RFID, and some sensors based on chipless RFID technology have been developed [34,35,36,37,38,39,40]. These chipless sensors show not only low cost but also high reliability even if these sensors work in harsh environment. However, the chipless RFID has a lack of commercial standard and needs a dedicated reader to interrogate the tag [41,42]. Importantly, the reader in such a system must undertake all interrogation and signal processing tasks due to the lack of intelligence of the tag and weakness of the returned ultra-wideband (UWB) signal. So far, the only commercially available chipless RFID tag is the surface acoustic wave (SAW) tag (developed by RF SAW) [43]. The wireless communication of Bluetooth has further read range than NFC, which can reach 10–100 m with the capability of low-power consumption. However, compared with NFC, Bluetooth technology needs a battery to power because it has no capability of energy harvesting. With the development of smartphones, NFC technology has been widely used to allow people to integrate their daily-use loyalty cards, credit cards, etc., due to its high security. Moreover, the cost of NFC readers is usually lower than that of other RFID devices. In the power available aspect, NFC technology can use batteries to transmit energy. However, NFC tags in the passive mode can harvest energy from the readers to power up the external electronics. Many sensitive NFC IC with the ability of energy harvesting have been available in the market, and NFC sensors based on energy harvesting have many applications, which can facilitate the development of low-cost and green energy wearable devices [44,45].

### 2.2. Commercial NFC Chips with Energy Harvesting

Power-consumption issues have always played an important role in the development of wearable devices. Batteries require to be strictly managed due to their many harmful ingredients. Thus, low-cost and green energy electronics attract wide attention and are suitable for the concept of green social development. Lazaro et al. reported a survey about the NFC sensors with the ability of energy harvesting, which introduced the working principle and applications of these NFC sensors [46]. Energy harvesting can be obtained from many ways (e.g., vibrations, electromagnetic sources, light, acoustic, airflow, heat, and temperature variations) [47]. In the passive or semipassive mode, NFC tags can collect energy from the magnetic field generated by readers to power up the external electronics. Some advanced NFC chips with capability of energy harvesting produced by the most important NFC IC manufactures have been available in the market, and some applications integrating NFC sensors with capability of energy harvesting have been introduced [48,49]. Table 2 shows some representative NFC IC information.

### 2.3. NFC Antenna Design

The design (e.g., size and shape) of NFC tags antenna is a very important aspect for the capability of energy harvesting. The size of loop coils is the key decision. A suitable size of loop coils determines its Q factor and read range. Here, Hirayama et al. reported a study about the coupling between two circular loop antennas to research the importance of antenna size [50]. Through analysis in literature, the coupling factor K is a function of radius r_1_ and r_2_ between the two radii for different axis distances *X* of two loop coils. The optimum case is when the two circles have approximately the same radius, which is *r*_1_ ≈ *r*_2_. Although in practice the effects of device size on the NFC antennas require to be considered, the radius between two loop coils should be the same, possibly, and this result is also suitable for other situations. In terms of NFC antennas shapes, special design for some applications can improve the antennas performance. Most antennas are designed in a circular shape. Different from this circular shape, recently, Kim et al. reported a kind of miniaturized flexible electronic system with wireless power and near-field communication capabilities, in which the shapes of antennas are elliptical [51]. In this work, the NFC antenna adopts a two-coil layout mode with coil diameters of 5.8 and 7.04 mm, respectively. In order to keep the high Q factors and resonance frequencies near 14 MHz, each of the coils in the dual-coil layout consists of copper traces with nine turns (18 µm thick). These NFC devices adopt elliptical shapes with major axes oriented parallel to the base of the nail. The results of these NFC devices with elliptical shapes, in which the ratios *b*/*a* = 1.21, 1.44, and 1.69, are simulated and tested. Although the amplitude of the phase and resonance frequencies decrease a little, it has no essential effects on the Q factors and resonance frequencies of the antennas. It can be seen from the above that a reasonable coil design will improve the overall performance of the NFC antennas in practical applications.

Additionally, the choices of materials can also improve the performance (e.g., Q factors and read range) of NFC antennas in some cases. Currently, most NFC antennas have been installed on the phone batteries, which may cause performance degradation due to the eddy current in the battery pack of a mobile handset. Some research works about a very thin ferrite sheet with a high relative permeability (*µ* ≈ 200) have been introduced to avoid the antennas losses [52]. Recently, Lee et al. reported a kind of NFC antenna design for low-permeability ferromagnetic material [53]. They proposed a novel structure of an NFC antenna with ferrite-polymer composites. Compared with other antennas, these NFC antennas with ferrite-polymer composites are more durable and have lower power consumption. The load modulation performance of the proposed NFC antenna increased over 65%, and its read range increased over 23%. Another strategy is utilizing the non-metallic materials to replace the metal materials for the NFC antennas. Graphene monolayers have attracted great attention due to their great capabilities of high conductivity, stretchability, and chemical resistance. “Graphene (G) paper” produced by carbon-based materials with high anisotropic 2-dimensional shape and good processability has been demonstrated in recent works [54,55]. In order to apply the “G paper” in commercial electronics, Scidà et al. reported an NFC device based on highly flexible, carbon-based antennas composed of stacked graphene multilayers [56]. In this work, the NFC antennas produced by the graphene with a high value of conductivity (4.20 × 10^5^ S/m) are tested before and after hundreds of thousands of bending cycles at bending radii of 45 and 90 mm. During bending, the self-resonance frequency and inductance peak show minimal variation and the resistance at 1 MHz changes from 33.09 to 34.18 Ω, outperforming the standard of commercial metallic antennas. Moreover, in actual applications, these proposed NFC antennas show better performance in the exchanging of data between smartphone and commercial NFC readers than the metal antennas. These proposed NFC antennas are achieved on different substrates and can be cut into different shapes without degradation of performance. Thanks to its flexible antennas, this proposed NFC tag can be used into the wearable devices. Textile is another representative material for NFC antenna fabrication. The e-textile has been used in wearable electronics due to its main advantages being easy and comfortable wearing. Ruben et al. reported a research work about the design and performance analysis of the textile spiral antenna for wearable applications [57]. In this work, the textile antenna has been designed with a square shape, and its line width is close to the conductive threads’ (between 0.1 to 0.6 mm). The result shows that the resonant frequencies of textile antennas are shifted up 0.8 to 1 MHz compared with the conventional antennas.

Another aspect that needs attention is that the detuning of tag antenna reducing wireless power transfer will occur when mobile phones are close to NFC tags. In order to study the effects of the detuning, recently, Boada et al. reported a kind of NFC sensors with energy harvesting [47] and introduced the effects of the detuning. The Q factors of NFC antennas can be obtained from Q = Im (Z) / Re (Z). In order to calculate the quality factor of whole transponder (Q_T_), a modified VNA set-up with an external amplifier and a reflectometer was used to characterize the tag under similar power conditions to the actual operation with a reader [58]. We found that the voltage-dependent parasitic capacitance increased. The Q factors of NFC antennas and input voltage decreased, and the bandwidth increased with the increase of distance. The resonance frequency tended to decrease due to the variation of the chip capacitance. Thus, the maximal distance between the tag and the reader to harvest the tag is 2 cm in the best of the two analyzed cases. 

### 2.4. Manufacturing Techniques

Manufacturing on soft substrates remains the challenge to fabricate wearable sensors and electronics. Some new manufacturing strategies have been adopted to develop electronic systems [59]. Currently, lithographic processes and printing are two prominent fabrication methods to produce electronics on soft substrate. Lithography is the most mature method to fabricate electronics systems with high controllability and precision [60,61,62]. However, lithography techniques are high-cost because they involve expensive equipment, strict working environments, and usually complex processes [63]. Compared with lithographic processes, printing technologies are more effective and attractive due to their excellent properties in simplifying processing steps, reducing materials wastage, low-cost, and simple patterning techniques. A review summarizes some recent printing techniques used to manufacture electronic components [64]. Here, we focus on the screen printing and inkjet printing technologies and discuss their potential and drawbacks in the manufacturing of NFC antennas.

**Figure 2:**
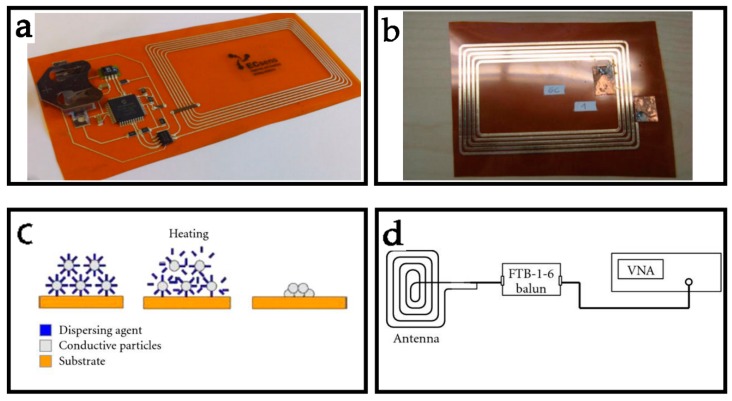
Printing technologies for NFC antenna design. (**a**) Screen printing for NFC antennas. Reprinted with permission from ref. [53]. (**b**) Printed prototype. Reprinted with permission from ref. [70]. (**c**) Sintering process. Reprinted with permission from ref. [70]. (**d**) Measurement set up. Reprinted with permission from ref. [70].

Screen printing is the most popular approaches to produce large area of electronic systems, with great simplicity, affordability, speed, and adaptability. When printing plate is printed, ink is transferred to substrates (e.g., paper and ceramics) through the aperture of stencil by a certain pressure, forming an image or a character. Salmerón et al. reported a research work of screen printing’s properties and printability (Figure 2a) [65]. As the Table 3 shows, screen-printed inductors in this work with lower mesh densities have better Q factors due to their greater thickness and corresponding lower sheet resistance. Meanwhile, in manufacturing industries, inks and conductive metal pastes present a growing demand due to the potential reduction of the manufacturing cost and processing steps. A kind of copper ink has been used to fabricate conductive electrodes and NFC antenna by screen printing [66]. In this work, the inductance of copper ink antenna is similar with the silver-paste antenna and its return loss measured −16.8 dB, which is lower than silver-paste antennas (−8.8 dB). Compared to copper-etched antenna, copper ink antenna has wider usable range of frequency band. However, the Q factor of copper ink antenna is too low to be used than conventional copper-etched antenna (about 7.8) due to its small thickness. In order to solve drawbacks of low Q factors, the multiple printing of the copper ink is required, or the copper ink should be modified for enabling thicker printing. However, metallic nanoparticles are also frequently used in conducting inks used for printing antennas, which can interact with atmospheric water or oxygen, causing the antenna to oxidize and degrade more rapidly than bulk metals. In many applications, as for example in wearable electronics, antennas have to withstand harsh chemical environments and therefore they have to be corrosion-resistant.

Inkjet printing is one of the most promising techniques that could potentially revolutionize large area and organic electronics fabrication, and its advantage for the fabrication of antennas is that the thickness of the printed coil can be controlled. Inks made of silver conductive nanoparticles have been widely used in inkjet printing to print electronics on the substrates [67,68,69]. As a consequence, input resistance and Q factors can be tuned as long as skin depth is not surpassed while keeping the same inductance levels. In order to examine the potential of inkjet technology for the NFC antennas’ fabrication, Ortego et al. reported an analysis of an inkjet-printed planar coil antenna, in which the substrate material is Dupont Kapton polyimide film 127 μm thick and the conductive ink is SunTronic Jettable Silver U5714 ink (Figure 2b,c) [70]. Once the inkjet-printed antenna is finished, the thickness can be measured by A KLA-Tencor P-16+ Profiler. As long as learning the number of printing layers, the thickness of each layer can be calculated. The corresponding Q factor and impedance of NFC antennas can be calculated by a standard VNA (Figure 2d). If the thickness remains under 40 µm, a Q value up to 90 can be theoretically achieved. Thus, inkjet printing is proved to be a promising technology for the fabrication of the NFC antennas, and due to its low-cost manufacturing process and substrate flexibility, it is a perfect candidate for the implementation of wearable NFC terminals. However, a remaining challenge is how to improve the conductive of inkjet-printed patterns for the antennas. The conductivity of conductive inks needs to improve, and the inkjet process should increase its thickness deposition capacity.

## 3. Wearable NFC Sensors for Healthcare

Recently, wearable electronic devices with different types of NFC sensors have been designed to acquire and transmit human’s physiological information instantly due to their capabilities of real-time monitoring and low-power consumption [71,72,73,74]. In this section, we will summarize the latest development in two types of wearable NFC sensors, which have biophysical and biochemical basis, representatively. Some of NFC sensors are represented in the Table 4.

### 3.1. Biophysical Signals Monitoring

**Figure 3:**
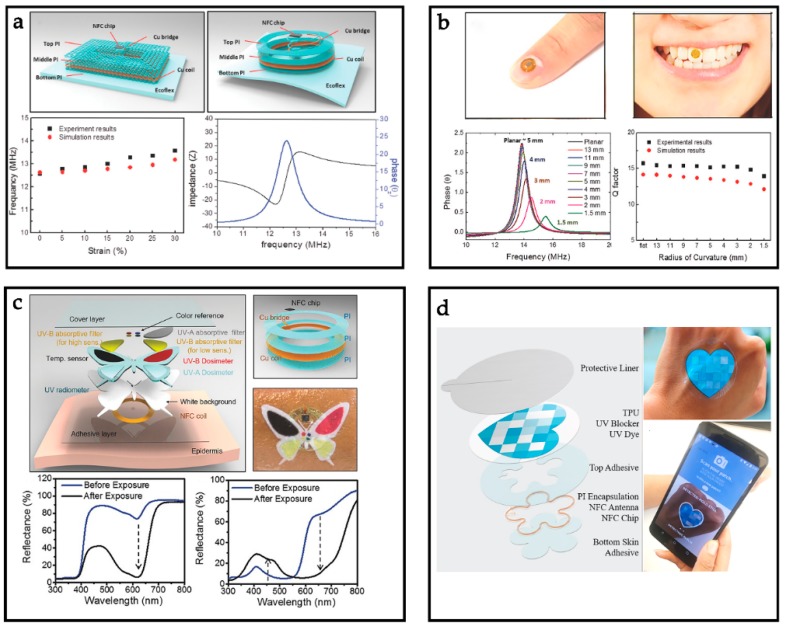
Representative examples of biophysical signals monitoring. (**a**) Exploded-view schematic illustrations of each layer of the skin-mounted NFC devices with rectangular coil. Reprinted with permission from ref. [83]. (**b**) Picture of a device on a tooth and fingernail. Reprinted with permission from ref. [51]. (**c**) Wearable NFC sensors attached onto teeth and fingers. Reprinted with permission from ref. [84]. (**d**) Construction of the UV sensor. Reprinted with permission from ref. [75].

The information of our health status can be obtained from the physical signals (e.g., pulse, heart rate, and body temperature) produced by the human body [80,81,82]. Recently, Kim et al. reported an NFC device that has an ultrathin construction, ultralow modulus, and can adapt to the changes of the skin without appearing to fall off (Figure 3a) [83]. In this work, epidermal NFC devices with NFC chips (M24LR04E, ST Microelectronics, 50 µm thick) are designed into rectangular (20 mm × 14 mm) and circular (16 mm φ) shapes, respectively. The rectangular coil adopts 19 turns of copper traces (5 µm thick and 76 µm wide) with the filamentary serpentine structure to maintain stretchability. The circular coil contains 15 turns of copper traces with similar thicknesses and widths but without the filamentary serpentine structure. The resonant frequencies of the two coil shapes are 12.9 MHz and 12.53 MHz, respectively, and the Q factors of these NFC antennas are 8 for the rectangular shapes and 14 for the circular coil, which meet the Q factor standards of commercial coils at similar frequencies. The result shows that the Q factors of these two proposed coils present no obvious change for deformations up to 30%. Meanwhile, when ensuring that wearable devices can adhere well to the surface of skin in daily activities, it is also required to endow wearable devices with the capacity to maintain communication ability at all times (Figure 3b). The group of Rogers also reported a kind of miniaturized flexible electronic system that can be developed with many other types of biosensors and electronic implants, increasing wearable NFC sensors’ great potential for development [51]. Here, commercial NFC chips (NTAG216, M24LR04E) with energy harvesting are used with the 5.8 mm and 7.04 mm diameter coils, respectively. The NFC antenna is designed with two-layered coils, which are designed into elliptical shapes to maintain the Q factor and the resonance frequencies. With the increase of the aspect ratio *b*/a, the resonance frequency and the amplitude of the phase decrease only slightly, and the Q factors remain almost unchanged. Araki et al. developed a new device based on the NFC system mentioned in [51] for an epidermal UV colorimetric dosimeter with the method of colorimetric chemistry (Figure 3c) [84]. A temperature sensor can measure the UV dose accurately since it was integrated into this device to provide capabilities in colorimetric temperature sensing. The NFC system not only endows the wearable devices with excellent stretchability but also allows the device to maintain good performance under bending conditions, even under a 30% strain condition. Shi et al. reported an UV patch based on the same NFC system, in which the photosensitive material changes color under illumination (Figure 3d) [75]. The photosensitive material corresponds to the intensity of the light and is analyzed by a smartphone. This device is very stable and has been tested to work even in long-term water or with sunscreen.

It is a great improvement that sensor arrays integrated in all parts of body would help us know the physiological mapping [85,86]. Recently, battery-free, wireless sensors for full-body pressure and temperature mapping were introduced [21]. The passive NFC tags adopt chips with energy harvesting capability and rely on the ISO/IEC 15693 standard, in which their read range can reach tens of centimeters. This device contains three main electronics: NFC antennas, temperature sensing, and analog-to-digital (A/D) conversion (ams AG; NFC die SL13A, 100 mm thick, 2.38 mm × 2.38 mm). NFC tags attached to the body are used for power transfer and data communication to a central acquisition/control system with a remote reader. The sensors were placed throughout the body to monitor the temperature and pressure changes and provide a function of spatiotemporal mapping of physiological processes. These wireless and battery-free epidermal electronics are designed to have an accurate measurement of the thermal properties, which can measure the body temperature regulation, wound healing, and other clinically relevant parameters.

The heart rate is also of great significance in medical care, and the abnormal heart rate usually reflects whether the heart is healthy [87,88,89,90,91]. Therefore, Lee et al. reported a new type of wearable and disposable cardiac biosensors (WISP) to monitor cardiac signals during daily activities, sleeping, and relaxing [92]. The WISP microcontroller optimizes the power consumption using an NFC system with the energy harvesting. When the smartphone is close to the WISP device, the NFC chips can collect additional wireless power from NFC-enabled smartphones [76,93,94]. In addition, when sensors collect information, NFC system will transmit this data to smartphone. The device can be worn not only on the body surface but also implanted in the human body to optimize the more difficult treatments in traditional medicine.

### 3.2. Biochemical Signals Monitoring

Wearable electronics collect physiological information from secretions (e.g., sweat, saliva, and tears) on the surface of the body, predicting disease occurrence and progress [95,96]. Here, some representative wearable electronic devices based on the NFC sensors will be introduced briefly to demonstrate how to predict the condition of the disease through detecting secretions.

**Figure 4:**
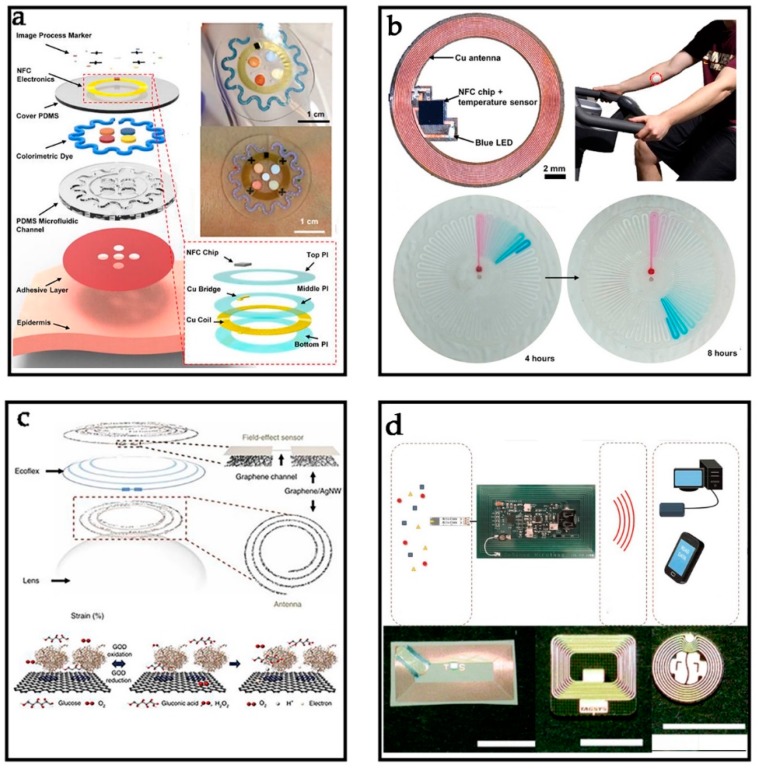
Applications for Biochemical signals monitoring: (**a**) Schematic illustration of an epidermal microfluidic sweat monitoring device and an enlarged image of the integrated near-field communication (NFC) system. Reprinted with permission from ref. [60]. (**b**) The NFC electronics consist of a magnetic loop antenna, NFC chip with an on-board temperature sensor, LED, and passive components. Reprinted with permission from ref. [22]. (**c**) Schematic of the wearable contact lens sensor, integrating the glucose sensor and intraocular pressure sensor. Reprinted with permission from ref. [102]. (**d**) Tears analysis with paper-based microfluidic system. Reprinted with permission from ref. [103].

Traditionally, physical examination was performed by detecting blood, which is an invasive detection method in medical treatment; moreover, this method cannot track in situ performance due to the blood sampling and post-mortem analysis [97,98]. Secretions of the human body contain several pieces of significant chemical information, providing a new idea to non-invasive detection, and sweat is the most viable liquid [99,100,101]. Recently, Koh et al. reported a new type of electronics to collect sweat of skin surface (Figure 4a) [60]. The ultrathin NFC electronics mentioned in the literature [83] have been used to endow this proposed device with stretchability, even under a 30% strain condition. The external electronics integrating with NFC systems have been utilized to launch image capture and analysis software. The sweat containing substances (e.g., chloride, lactic acid, and glucose) is collected into the device through the embedded microfluidic channels, and then, it will react with the colorimetric reagents in each chamber. Of course, this detection concept could be applied into many other wearable devices to collect sweat, increasing the ability of mastering our body information to diagnose diseases (e.g., diabetes, cystic, and fibrosis) (Figure 4b) [22].

Tears, which are the biochemical analysis of body fluids, contain many substances (e.g., water, glucose, lactic acid, and lactate) and have usually been evaluated as the direction for the system disease, including glaucoma, diabetes mellitus, and cancer [104,105]. Recently, Kim et al. reported a multifunctional contact lens sensor developed for monitoring glucose levels in tears and measuring the intraocular pressure (Figure 4c) [102,103,106]. The main challenge is how to provide adequate power for the sensors. Here, an RFID tag with energy harvesting capability has been integrated on glass to transmit energy to sensors (Figure 4d). Experiments on rabbit and bovine eyeballs showed its reliable performance, which demonstrated great application prospects in the medical field. Meanwhile, Rahimi et al. developed a pH-sensing system to monitor wound healing and determine the likelihood of early infection [78]. Alkaline environment provides a facilitating effect for microorganism, which may cause wound infection, and low pH is beneficial to inhibition of microorganism growth. The sensor is interfaced to an NFC SL13 chip from AMS with a buffer amplifier (AD8603, Analog Devices Inc., Norwood, MA, USA). The pH sensor exhibits a linear sensitivity of −55 mV/pH and stable performance under mechanical bending in a pH range of 4 to 10. This pH sensor could help us clean up the wound regularly with the information transported by the NFC.

## 4. Other Representative Applications of NFC Sensors 

Although the focus of the NFC sensor development has been on wearable devices, there are still some other representative applications (e.g., food safety, and botany). In this section, we summarize recent progress of NFC sensors for the other applications in people’s daily life.

**Figure 5:**
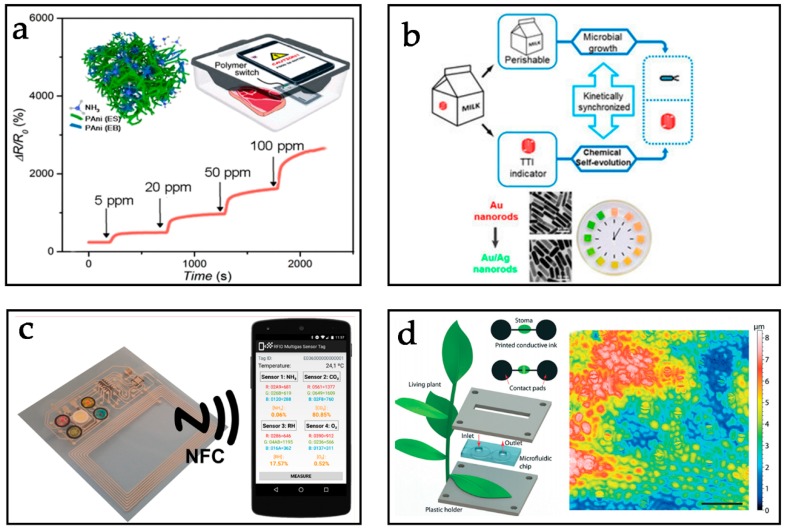
Other representative applications of NFC sensors. (**a**) Highly sensitive, printable nanostructured conductive polymer wireless sensor for food spoilage detection. Reprinted with permission from ref. [23]. (**b**) Schematic illustrating the expected “biochemo” synchronicity. Reprinted with permission from ref. [112]. (**c**,**d**) Plants growth monitoring. Reprinted with permission from ref. [24].

Food safety has always attracted great attention since humans are unable to live with lack of food [107,108,109,110,111]. Therefore, it is very important to develop an efficient, simple, and easy-to-operate measurement. Recently, Ma et al. reported a new NFC tag (Figure 5a) for food spoilage detection with the advantages of highly sensitive, printable nanostructured conductive polymer [23]. These passive NFC tags are manufactured through inkjet printing process and are powered wirelessly by generating a voltage across their antenna coil when receiving a radio frequency field emitted by mobile devices. The NFC tags with mode of “Off-to-On” are used as a switch. When detecting low-resistance polyaniline (PAni), the tags present “Off” state and are unreadable due to the mismatch between tags and readers. The switch of tags turns “On” state when contacting with total volatile basic nitrogen (TVBN), which can be used to detect the meat spoilage. Recently, Escobedo et al. reported a passive NFC tag with capability of energy harvesting and data transmission for multigas sensing, which contains four gas sensors (Figure 5b) [78]. The NFC chip (SL13A) with energy harvesting capability collects electromagnetic energy to power up the tag sensing area. What is more, an Android software was developed on the NFC-enabled smartphone to allow in situ measurement of the gas sensors. The NFC chemical gas sensor uses a white LED lamp to provide energy and reads the light response through a high resolution digital color detector. By calibrating the chemical gas sensors, the gas concentration can be measured quickly, and the data can be transmitted to the smartphone. Meanwhile, Potyrailo et al. reported a kind of battery-free RFID sensors for food safety (Figure 5c) [79,112]. These passive RFID tags, posted outside of the milk carton to avoid contaminating food, can analyze changes in the dielectric constant from the environment. When printed on the paper substrate, the RFID tags combined with inductor and capacitor can monitor the humidity levels of the package of milk, which are the direct testing standards for milk quality. 

Water, light, and nutrients (e.g., nitrogen, phosphorus, potassium, calcium, and magnesium) are essential factors in plant growth, therefore, continuously monitoring these factors is very important for the development of botany [113]. Water is very important for plant growth, and an unsuitable amount of water will lead to the abnormal growth of plants. Recently, Boada et al. reported a battery-less soil moisture measurement system [48]. This system is based on an NFC sensor with energy harvesting capability to measure the volumetric water content, temperature, and relative humidity. The NFC IC (M24LR04E-R) with energy harvesting capability is used in the NFC tags to power up electronics. The data collected by NFC tags can be stored into the cloud to keep monitoring the soil conditions. The ambient temperature sensors using an I^2^C temperature sensor (LM75A) and humidity sensors using HIH-5030 humidity sensor are integrated in the NFC tags to monitor the environment. The operation of external electronics needs less than 1 mA at 3 V. When the information of soil is collected by the sensors, the data will be shown on the smartphone. Additionally, Koman et al. reported an amicrofluidic-printed electro-mechanical sensor for persistent-drought monitoring (Figure 5d) [24]; a stomatal electro-mechanical pore size sensor (SEMPSS) and a microfluidic channel have been developed to trace single stoma-aperture dynamics for persistent drought monitoring of plants without damaging the stomata. The printed electronics (e.g., RFID systems, electrochemical and logic circuits) can endow the devices with more complex capabilities, enabling the monitoring and engineering of new plant functions.

## 5. Conclusions

Although near-field communication technology was introduced in 2000, it was only designed to be applied to other fields (e.g., healthcare and food safety) until recent years. Wearable devices combined with NFC sensors could be more comfortable and seamless to the touch of skin, and compared with traditional wearable electronics, they hold a distant prospect in a non-invasive medical method. Considering that many wearable electronics require a large battery supply for long-term, continuous monitoring, energy installations usually increase the devices’ dimensions, limiting the ability to touch with skin. Therefore, the development of a miniature battery transmission system is also an urgent problem to be solved. Meanwhile, the choice of sensor requires to be taken into serious consideration, and biosensor receives great interest due to its excellent sensitivity and reproducibility. Integrating a single sensor tag into a unified sensor array will help us with all-round monitoring and dynamic analysis; however, another question requiring consideration is how to eliminate challenges posed by high-density integration. Additionally, considering the widespread applications of such wearable devices in the future, the selection of biodegradable, non-toxic materials is particularly significant and may open a new frontier for bioanalyte analysis.

In this paper, we briefly discussed antenna design and manufacturing techniques of NFC sensors and some of their representative applications in healthcare, food safety, and botany. Combined with wearable technology, these NFC sensors provide continuous monitoring capabilities the traditional wearable sensor could not achieve. Through our summary above, we found that NFC sensors have wide applications, ranging from medical care and food detection to plants’ growth. We believe that with the continuous advancement and development of technology, NFC sensors could provide us with a better experience, which may open up opportunities for an even broader range of applications.

## Figures and Tables

**Table 1:** Comparison of Several Wireless Communication Technologies.

Feature	NFC	Bluetooth	UHF RFID	Chipless RFID
Reader cost	Low, smartphone	Low, smartphone	High, $1000–$2000	High, no commercial
Read range	1–2 cm for proximity cards with energy harvesting, 0.5 m for vicinity cards	10–100 m	Up to 15 m with inlay tags with 2 dBm read IC sensitivity. Up to 3.m UHF sensors (with −9 dBm read IC sensitivity). Up to 30 m BAP.	<50 cm frequency coded 2–3 m, time-coded UWB
Universal Frequency regulation	Yes, ISM	Yes, ISM	No, by regions	No, often used UWB
ID rewritable	Yes	Yes	Yes	No
Energy harvesting	Approx. 10 mW	NO	Few µW	NO
Tag price	Low	High	Low	Moderate
Memory capacity	<64 kilobits	Several kilobytes depending on the microcontroller	96 bits EPC, typically 512 bits for users (<64 Kbytes)	<40 bits

**Table 2:** NFC IC with the capability of energy harvesting (ADC—analog-to-digital converter; SPI—serial peripheral interface; UART—universal asynchronous receiver-transmitter).

NFC IC	Energy Harvesting Maximum Sink	ADC	Bus	Comments
M24LR04E-R	6 mA/3 V	Yes	I^2^C	ISO 15693
GT23SC6699-1/2 Giantec Semiconductor	NA/3.2 V	No	I^2^C	ISO 15693
SIC4310, SIC4340, SIC4341 Silicon Craft	10 mA/3.3 V	No Yes	UART	220 bytes EEPROM ISO 14443A
SL13 AMS AG	4 mA/3.4 V	Yes	SPI	8 kbit ISO 15693
MLX90129 Melexis	5 mA/3 V	Yes	SPI	4 kbit ISO-15693

**Table 3:** Inductance (*L*) and quality factor (Q) at 13.56 MHz.

Substrate	Technique	*L* (µH)	Q factors
PI	Screen printing 90 T/cm	5.15 ± 0.44	5.13 ± 0.64
	Screen printing 140 T/cm	5.08 ± 0.08	2.51 ± 0.08
PET	Screen printing 90 T/cm	5.09 ± 1.20	3.38 ± 0.68
	Screen printing 140 T/cm	5.28 ± 0.31	2.50 ± 0.02

**Table 4:** References of NFC sensors.

Reference	Chips	Passive	Sensors Functions
[51]	NTAG216 M24LR04E	Yes	Biosensors and electronic implants
[75]	SL13A, ams AG	Yes	Measuring the UV dose
[21]	Sl13A, AS62x0	Yes	Measuring the temperature and pressure
[76]	AMS SL13A, AMS Inc	Yes	Monitor the thermal characterization of skin
[60]	M24LR04E	Yes	Analysis sweat
[22]	AMS SL13A	No	Sweat collection for biomarker analysis
[77]	AMS SL13	No	Wound pH monitoring
[23]	NFC-WISP	Yes	Food safety monitoring
[78]	SL13A	Yes	Gas monitoring
[79]	M24LR	Yes	Soil Moisture Measurement
